# GC-MS profiling and assessment of antioxidant, antibacterial, and anticancer properties of extracts of *Annona squamosa* L. leaves

**DOI:** 10.1186/s12906-020-03029-9

**Published:** 2020-10-06

**Authors:** Rawan Al-Nemari, Abdulrahman Al-Senaidy, Abdelhabib Semlali, Mohammad Ismael, Ahmed Yacine Badjah-Hadj-Ahmed, Abir Ben Bacha

**Affiliations:** 1 Protein Research Chair, Department of Biochemistry, College of Science, King Saud University, Riyadh, Kingdom of Saudi Arabia; 2 Groupe de Recherche en Écologie Buccale, Faculté de Médecine Dentaire, Université Laval, Québec, Canada; 3 Advanced Materials Research Chair, Department of Chemistry, College of Science, King Saud University, Riyadh, Kingdom of Saudi Arabia; 4 Department of Biochemistry, College of Science, King Saud University, Riyadh, Kingdom of Saudi Arabia; 5 Laboratory of Plant Biotechnology Applied to Crop Improvement, Faculty of Science of Sfax, University of Sfax, Sfax, Tunisia

**Keywords:** *A. squamosa*, Phytomedicine, Bioactive compounds, Antioxidants, Antibacterial, Apoptosis

## Abstract

**Background:**

The research and application of plants in food supplements and drugs have attracted great interest. This study aimed to examine the efficiency of several solvents for the extraction of the main compounds from *Annona squamosa* leaves and to evaluate the antioxidant, antibacterial, and anticancer activities of these extracts.

**Methods:**

Gas chromatography-mass spectrometry was used to screen the bioactive compounds of *A. squamosa* methanolic extract*.* The free radical, hydrogen peroxide, and nitric oxide scavenging activities of the extracts were investigated. Furthermore, MTT, nuclear staining, LDH, and monolayer wound repair assays were performed to evaluate the potential anticancer activity of the extracts in colon cancer cells while the antibacterial activity was tested by using a well diffusion assay.

**Results:**

*A. squamosa* leaves extracts were found to contain several bioactive compounds, of which the majority were sesquiterpenes (C_15_H_24_). These extracts exhibited strong antioxidant activity and antibacterial potency against both gram-positive and gram-negative bacteria. Different *A. squamosa* leaves extracts displayed remarkable antiproliferative, cytotoxic, antimigration, and apoptotic activities in colon cancer cells.

**Conclusions:**

*A. squamosa* leaves contain major bioactive compounds that inhibit the growth of several types of bacteria and colon cancer cell lines, which demonstrated their efficacy as an alternative source of antibiotics and for the development of novel drugs for colon cancer therapy.

## Background

One of the most studied topics over the last two decades among biologists worldwide is oxidative stress as it is commonly associated with several diseases including autoimmune and inflammatory diseases [1, 2]. Oxidative stress is a signal that reflects the overwhelming production of reactive oxygen and nitrogen species (RONS) that exceeds the antioxidant capacity within a cell. Prolonged oxidative stress can damage cellular building blocks, disrupt cellular signaling, and induce the release of inflammatory signaling molecules [3, 4]. Indeed, RONS-mediated oxidative stress has been found in the initiation, development, and progression of cancer. Cancer is the leading cause of death in economically developed countries and the second leading cause of death in developing countries, making it a serious global problem [5, 6]. Despite the availability of effective treatment options for the early stages of cancer, such as chemotherapy, surgery, radiotherapy, and hormone therapy, their use is costly and remains limited in the later stages of cancer. They can cause serious side effects in the patient and pose a real problem for public health [7, 8]. In addition, one of the most critical aspects of cancer therapy that faces scientists and doctors is resistance to treatment, which can occur after prolonged treatment with the same drug, reducing the level of RONS and inducing resistance to apoptosis [2, 3]. Currently, researchers investigate second-line treatments, which could be natural products, for use in addition to chemotherapy or even as chemo-preventative agents [9]. Recent statistics have shown that approximately 85% of people use plant extracts to treat various diseases in the country based on their healthcare needs [10]. Many studies have reported the critical roles of natural plant extracts in the development of new anticancer drugs. They have emerged as potential compounds for use as adjuvant or complementary anti-cancer drugs with fewer side effects [11].

The antibiotic revolution has decreased the spread and severity of many minor diseases. However, due to the increasing number of microbial infections and the uncontrolled use of antibiotics, antibiotic resistance becomes a global public health threat. In 2012, the World Health Organization called for urgent corrective action, including the identification of new therapeutic agents, resulting in the increased frequency of microbial resistance and its association with serious infectious diseases [12]. Traditional herbal medicines are reported to possess antimicrobial effects. Thus, researchers have become more interested in the antimicrobial activity of natural products [13–15]. Through the use of natural antimicrobial compounds, the opportunity for bacteria to acquire resistance is minimized, and bacteria can be targeted via several mechanisms [16].

*Annona squamosa* L. (*A. squamosa*) is a small group of edible fruits belonging to the Annonaceae family and *Annona L.* genus, commonly known as custard apple. It is a native species found in tropical and subtropical regions worldwide. Traditionally, all parts of *A. squamosa* are used to treat different diseases in many countries, including India and China. For example, the seed powder is used to eliminate head lice, the leaves are used as a poultice to heal boils and ulcers, and the part of the fruit are used a sedative for the heart, to alleviate vomiting, and to treat cancer [17, 18]. In recent decades, several studies have reported that different parts of *A. squamosa* contain several bioactive compounds, such as acetogenin, alkaloids, steroids, terpenoids, saponins, and phenolics, which exert various biological activities [18, 19]. However, fewer studies were conducted on *A. squamosa* L. than other species within the same genus; many fewer studies focused on the leaves than other parts, and these mostly explored the antioxidant and antibacterial activities. This study aimed to investigate the constituent bioactive chemicals and the antioxidant, anticancer, and antibacterial activities of three different extracts of *A. squamosa* leaves.

## Methods

### Plant material

*A. squamosa* L. leaves were collected from a local plant nursery in Ta’if, Saudi Arabia, in December 2016. The identification and authentication was performed by Ibrahim Al-Dakhil, an agronomist, then confirmed by the Department of Botany and Microbiology, College of Science, King Saud University. A voucher specimen (KSU-No. 12068) was deposited at the Herbarium of the college of science, KSU. The plant leaves were then washed with running tap water until clean, shade-dried for 7 days, crushed into small pieces, and powdered by using an electric blender. The obtained powder was stored at − 20 °C until subjected to further extraction procedures.

### Extraction of plant material

Three different extracts were prepared from dried *A. squamosa* leaves powder by using three solvents of different polarity: methanol, acetone and water. The methanol and acetone extracts were prepared by macerating the dried powder with methanol or acetone (1:10 w/v) for 48 h. The extracts were filtered using Whatman No. 40 filter paper (Whatman® Schleicher & Schuell, UK) and concentrated under a fume hood via evaporation at 18–21 °C (room temperature). For the aqueous extract, the dried powder was macerated with distilled water (1:10 w/v) for 48 h. After centrifugation at 10000 rpm for 10 min, the sediment was discarded, and the resulting aqueous fraction was filtered through filter paper and lyophilized by using FreeZone 4.5 Liter Benchtop Freeze Dry System (Labconco, USA). Finally, *A. squamosa* extracts were weighed to determine the extraction yields (%w/w) and stored at − 20 °C in light-protected sterile containers for further experiments. For use, each extract was dissolved in the initial extraction solvent.

### Total phenolic content

The total phenolic content (TPC) of *A. squamosa* leaves extracts was estimated spectrophotometrically using the Folin-Ciocalteau method [20]. For each sample, 0.3 mL (80 μg/mL) was mixed with Folin-Ciocalteau reagent (1.5 mL; diluted 10 times) and sodium carbonate (1.2 mL; 7.5% w/v). After incubation of the mixture for 30 min at room temperature, the absorbance was measured at 765 nm. The total phenolic content was expressed as gallic acid equivalents (GAE) in mg per g of dry material (mg GAE/g), using a standard calibration curve.

### Total flavonoid content

The total flavonoid content (TFC) of *A. squamosa* extracts was determined according to Loganayaki et al. [21]. First, an 0.25 mL aliquot of the extract (80 μg/mL) was mixed with distilled water (1 mL), followed by the addition of 5% NaNO_2_ solution (0.075 mL). After 5 min, 10% AlCl_3_ solution (0.15 mL) was added to the mixture, which was then incubated for 6 min. Finally, 4% NaOH (0.5 mL) was added and the volume was adjusted to 5 mL with distilled water. After incubation for 15 min, the absorbance was determined at 415 nm. The total flavonoid content was expressed as quercetin equivalents in mg per g of dry material (mg quercetin/g), using a standard calibration curve.

### Gas chromatography coupled with mass spectrometry

The chemical composition of the methanolic extract of *A. squamosa* leaves was investigated by using gas chromatography coupled with mass spectrometry (GC-MS). GC-MS analysis was performed using a Thermo Trace GC Ultra gas chromatograph coupled with a TSQ Quantum mass spectrometer (triple quadrupole). The mass detector was operated at 70 eV ionization energy, 0.132 s/scan in full scan mode, over the mass range of 40–500 Da. The chromatograph was equipped with a Thermo TR-5MS fused silica capillary column (length, 30 m; i.d., 0.25 mm; and film thickness, 0.25 μm). The stationary phase was 5% phenyl polysilphenylene-siloxane. The following oven temperature was increased from 40 °C to 300 °C between 0 and 10 min at a rate of 6 °C/min, with the injector temperature of 250 °C. Helium was used as the carrier gas, with a flow rate of 1 mL/min and a split flow of 25 mL/min, which corresponded to a split ratio of 25. The transfer line temperature was set at 250 °C. The compounds were identified through the comparison of their mass spectra with the reference mass spectra of several libraries, including Wiley Library 7n.1, the NIST (National Institute of Standards and Technology), and previously published literature data.

### Antioxidant activity

#### Free radical scavenging activity

The antioxidant activity of *A. squamosa* extracts was measured in vitro on the basis of the scavenging activity of the 2,2-diphenyl-1-picrylhydrazyl (DPPH) free radical, as previously reported by Narasimhan et al. [22]. First, 0.1 mM DPPH (1 mL) was mixed with 1 mL of various concentrations of plant extracts (10–100 μg/mL). The corresponding blank samples were also prepared, along with a control containing distilled water instead of the extract. The reaction was conducted in triplicate using L-ascorbic acid (2–100 μg/mL) for the standards. After incubation in the dark for 30 min, the absorbance at 517 nm was measured. The percentage of DPPH radical scavenging activity was calculated from the following equation:

$$ \mathrm{Scavenging}\ \mathrm{activity}\ \left(\%\right)=\left(\mathrm{absorbance}\ \mathrm{of}\ \mathrm{control}-\mathrm{absorbance}\ \mathrm{of}\ \mathrm{sample}\right)/\mathrm{absorbance}\ \mathrm{of}\ \mathrm{control}\kern0.5em \times \kern0.62em 100 $$

#### Hydrogen peroxide scavenging activity

To measure the hydrogen peroxide (H_2_O_2_) scavenging activity of *A. squamosa* extracts, the method of Ruch et al. [23] was used. Different concentrations (0.1–1 mg/mL) of 0.1 mL of each extract were treated with 40 mM H_2_O_2_ solution (0.6 mL) in phosphate buffer (pH 7.4). After 10 min, the absorbance of H_2_O_2_ was measured at 230 nm against a blank solution of phosphate buffer. The control was prepared by using distilled water instead of the extract and 0.1–1 mg/mL L-ascorbic acid was used for the standards. The percentage of H_2_O_2_ radical scavenging activity was calculated from the following equation:

$$ \mathrm{Scavenging}\ \mathrm{activity}\ \left(\%\right)=\left(\mathrm{absorbance}\ \mathrm{of}\ \mathrm{control}-\mathrm{absorbance}\ \mathrm{of}\ \mathrm{sample}\right)/\mathrm{absorbance}\ \mathrm{of}\ \mathrm{control}\kern0.5em \times \kern0.62em 100 $$

#### Nitric oxide scavenging activity

The ability of *A. squamosa* extracts to scavenge nitric oxide (NO) was assessed by the method of Garratt et al. [24]. An aliquot of 0.5 mL of 10 mM sodium nitroprusside (in phosphate buffer saline pH 7.4) was mixed with 1 mL of each extract (10–100 μg/mL). After incubation at 25 °C for 180 min, an equal volume of fresh Griess reagent was added. Griess reagent was prepared by mixing 1% sulfanilamide in 5% phosphoric acid and 0.1% naphthylethylene diamine dihydrochloride in distilled water. The corresponding blank sample, without sodium nitroprusside, and the control, containing phosphate buffer instead of extract, were also prepared, and gallic acid and L-ascorbic acid (10–100 μg/mL) were used for the standards. Finally, the absorbance at 546 nm was measured and the percentage of NO radical scavenging activity was calculated from the formula:

$$ \mathrm{Scavenging}\ \mathrm{activity}\ \left(\%\right)=\left(\mathrm{absorbance}\ \mathrm{of}\ \mathrm{control}-\mathrm{absorbance}\ \mathrm{of}\ \mathrm{sample}\right)/\mathrm{absorbance}\ \mathrm{of}\ \mathrm{control}\kern0.5em \times \kern0.62em 100 $$

#### Reducing power

The reducing power of *A. squamosa* extracts was investigated by using slight modifications to the method of Oyaizu [25]. The extracts (0.25–1 mg/mL) were mixed with phosphate buffer (2.5 mL, pH 6.6) and 1% potassium ferricyanide (2.5 mL), and incubated at 50 °C for 20 min. After the addition of 10% trichloroacetic acid (2.5 mL), the mixture was centrifuged at 650 rpm for 10 min. Subsequently, the upper layer of the mixture (2.5 mL) was mixed with an equal volume of distilled water and freshly prepared 0.1% ferric chloride (0.5 mL). Finally, the absorbance was measured at 700 nm; butylated hydroxytoluene (BHT) and L-ascorbic acid were used as the standards.

### Antiproliferative activity

#### Cell culture

Colon cancer cell lines (Lovo and HCT-116) were obtained from American Type Culture Collection (ATCC, USA) and cultured in Dulbecco’s Modified Eagle’s (DMEM) medium (Gibco, USA) supplemented with 10% fetal bovine serum (FBS; SIGMA, USA) and 2 × 10–3 v/v penicillin-streptomycin (composed of 31 g/L penicillin, 50 g/L streptomycin) in a humidified atmosphere with 5% CO2 at 37 °C. During cell culture, the medium was replaced every 2 days until 80% confluency was reached.

#### Cell viability assay

The anti-proliferative activity of the extracts was determined by using the 3-(4.5-dimethythiazol-2-yl)-2,5-diphenyl tetrazolium bromide (MTT) assay [26]. All extracts were dissolved in DMSO and subsequently diluted in culture medium. Briefly, the cells were seeded in 6-well plates (4 × 105 cells/well) and treated with various concentrations of the leaves extract (1 μg/mL, 10 μg/mL, and 100 μg/mL) for 24 H*. medium* was removed, the cells were washed twice with PBS and incubated with 100 μL MTT (5 mg/mL; Sigma, USA) for 4 h. Subsequently, the supernatant was removed, and 0.04 N HCl in isopropanol (500 μL) was added to each well. Finally, 100 μL of the reaction mixture was transferred to a 96-well plate and the maximum absorbance at 550 nm was detected by using an ELISA plate reader (X-Mark Microplate Spectrophotometer, Bio-Rad, USA). The experiment was conducted in triplicate. The percentage of cell viability was calculated from the following formula:

Cell viability (%) = (absorbance of test sample/absorbance of negative control) × 100.

#### Nuclear staining

The morphology of treated cells was assessed by using a Hoechst 33342 staining assay (H42) as previously described by Semlali et al. [26]. The cells were seeded in 6-well tissue culture plates (3 × 105 cells/well). After overnight incubation, the medium was removed, replaced with media, and the cells were treated with different concentrations of extracts and incubated for 24 h. The supernatant was then removed, the cells were washed twice with PBS and cold methanol was used to fix the cells for 15 min. These cells were washed twice with PBS and subsequently stained with 2 μg/mL Hoechst 33342 (Thermo Fisher Scientific, DE) for 15 min in the dark. The stained nuclei were washed twice with PBS and observed and photographed under Leica DM2500 & DM2500 LED optical microscopes (LEICA Microsystems, DE).

#### Cytotoxicity assay

The cytotoxicity of *A. squamosa* extracts on Lovo and HCT-116 cell lines was quantitatively assessed through the measurement of lactate dehydrogenase (LDH). The cells were cultured for 24 h in 6-well plates (4 × 105 cells/well). After overnight growth, the culture medium was removed and replaced by 1 mL of culture medium. Then, the cells were treated with various concentrations of the leaves extract (1 μg/mL, 10 μg/mL, and 100 μg/mL) for 24 h. To estimate LDH activity, 10 μL of the culture supernatant was transferred to a new 96-well plate and the enzyme reaction was conducted in accordance with the manufacturer’s instructions (LDH cytotoxicity colorimetric assay kit II, BioVision, USA). The cytotoxicity was calculated as a percentage by using the following formula:

Cytotoxicity (%) = (test sample – negative control) / (positive control – negative control) × 100.

#### Monolayer wound repair assay

Colon cancer cell lines were grown in 6-well plates. Wounds were made in a confluent monolayer of each well by using a 10 μL pipette tip. The cells were then treated with 50 μg/mL of the methanolic, acetonic, or aqueous extract and the wound closure was compared with that of untreated cells at 0, 6, and 24 h after wounding [27]. Digital photographs were captured with a LEICA DFC450 C digital camera (LEICA Microsystems, DE). The percentage of wound closure was calculated through the comparison of the wound areas before and after stimulation using the following formula:

Wound closure (%) = (initial scratch size – size of the scratch after an identified culture period) / (initial scratch size) × 100.

### Antibacterial activity

#### Bacteria and growth conditions

Based on their clinical and pharmacological importance, *Bacillus subtilis* (*B. subtilis*), *Staphylococcus aureus* (*S. aureus*), *Enterococcus faecalis* (*E. faecalis*), *Escherichia coli* (*E. coli*), *Pseudomonas aeruginosa* (*P. aeruginosa*), *Klebsiella pneumoniae* (*K. pneumoniae*), and *Salmonella typhimurium* (*LT2*) were selected for the evaluation of antibacterial activities of the *A. squamosa* leaves extracts. These bacterial strains were obtained from Botany and Microbiology Department, College of Science, King Saud University. The fresh culture of each microorganism was grown in Luria-Bertani media (Bio Basic, CAN), until McFarland standard 0.5 was reached. Subsequently, the suspensions were diluted with sterile 0.9% normal saline (1:100) to obtain 106 colonies forming unit/mL.

#### Agar well diffusion assay

The antibacterial activity of *A. squamosa* leaves extracts was assessed against different bacterial species by using an agar well diffusion assay following the method of Balouiri et al. [13]. The bacterial inoculum was uniformly spread by using a sterile cotton swab on a Mueller-Hinton agar plate (Becton Dickinson, USA). Then, a hole (diameter, 6 mm) was punched aseptically with a sterile tip and 100 μL of each extract solution (50 mg/mL in dimethyl sulfoxide; DMSO; Sigma, USA) was used to fill the wells. Reference commercial discs (30 μg Tetracycline; OXOID) were also conducted, and 100 μL DMSO, instead of extract, was used for the negative control. After incubation for 24 h at 37 °C under aerobic conditions, the zone of inhibition of bacterial growth was measured in millimeters. Experiments were performed in duplicate.

### Statistical analysis

Statistical analyses were computed by using SPSS software (Statistical Package for the Social Sciences; version 21 for Mac). The values were presented as the arithmetical mean ± standard deviation (±SD). The statistical significance of differences was evaluated by one-way analysis of variance (ANOVA) followed by Dunnett’s multiple comparison test. *P* ≤ 0.05 was considered to indicate statistical significance, whereas a value of ≤0.005 was considered to indicate a highly significant statistically difference to the relevant control.

## Results

### Determination of extraction yield, TPC, and TFC of *A. squamosa* leaves extracts

The weight (percentage yield) of the obtained dried crude extracts was calculated with respect to the initial amount of the dried powder; the values are presented in Table 1. It was observed that the water extract was obtained in the highest yield, followed by the methanolic and acetonic extracts. The highest TPC was found in methanol extract, followed by the acetone and water extracts. While the TFC of the acetonic extract was four times higher than that of the methanolic extract (7.1 ± 0.9 mg quercetin/g and 1.8 ± 0.1 mg quercetin/g, respectively); however, only trace amounts were present in the water extract (0.1 ± 0.05 mg quercetin/g) (Table 1).

**Table 1:** Percentage extraction yields obtained from *A. squamosa* extracts, total phenolic and flavonoids content, and the IC_50_ values for DPPH, H_2_O_2_, and NO scavenging activity

*A. squamosa* leaves extracts	Extraction yield (% w/w)	Total phenolic content (mg GAE/g)	Total flavonoids content (mg quercetin/g)	IC_50_		
				DPPH (μg/ml)	H_2_O_2_ (mg/ml)	NO (μg/ml)
Methanol	6.96 ± 0.68	282.1 ± 11.2	1.8 ± 0.1	51 ± 1.6	735 ± 49.5	12 ± 4.2
Acetone	5.84 ± 0.33	256.3 ± 12.5	7.1 ± 0.9	33.9 ± 4.8	516.7 ± 5.8	44 ± 5.7
Water	13.07 ± 0.24	16.9 ± 0.4	0.1 ± 0.05	98.3 ± 0.4	110 ± 14.1	81 ± 1.4

The results are presented as mean ± SD (*n* = 3)

### Identification and characterization of chemical compounds in *A. squamosa* leaves extracts

**Fig. 1:**
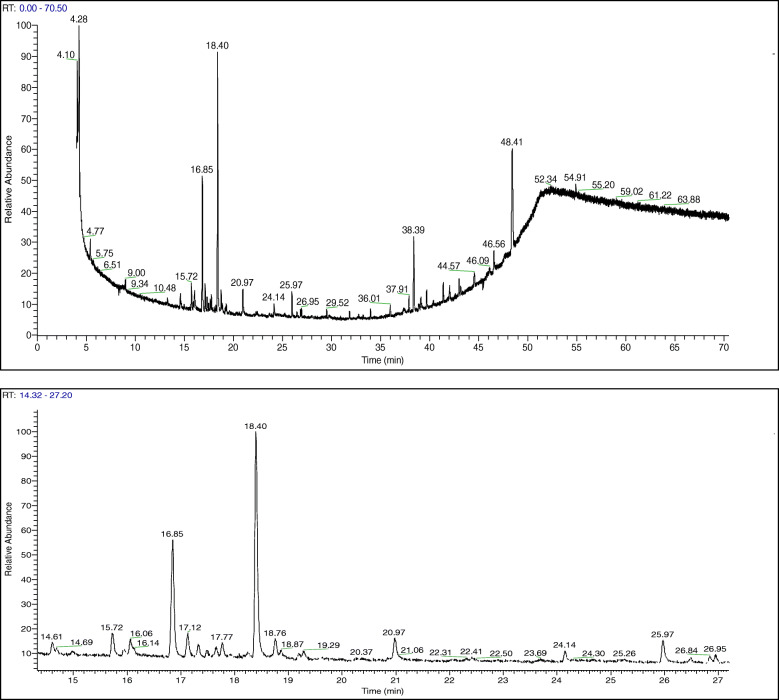
Gas chromatography-mass spectrometry profile of methanolic extract of *Annona squamosa* leaves. RT: retention time

The GC-MS profile of the methanolic extract of *A. squamosa* leaves is shown in Fig. 1. The main constituents identified in the extract are reported in Table 2. Most components were sesquiterpenic hydrocarbons, such as germacrene-D (22.01%), trans-caryophyllene (12.12%), bicyclogermacrene (2.80%), α-copaene (2.12%), and humulene (1.15%), as well as phytol (2.22%) and squalene (1.3%). Further investigation into the main constituents of the acetonic and aqueous extracts of *A. squamosa* leaves well be covered in the future.

**Table 2:** The major compounds identified in methanolic extracts of *A. squamosa* leaves; retention times (RT), classification, formula and molecular weight (Mw)

No.	RT	Proposed compound	Class	Formula	Mw	%
1	14.60	bicycloelemene	sesquiterpene	C_15_H_24_	204	1.28
2	14.69	d-elemene	sesquiterpene	C_15_H_24_	204	0.46
3	15.72	α -copaene	sesquiterpene	C_15_H_24_	204	2.12
4	15.94	β-bourbonene	sesquiterpene	C_15_H_24_	204	0.71
5	16.85	trans-caryophyllene	sesquiterpene	C_15_H_24_	204	12.12
6	17.48	α-amorphene	sesquiterpene	C_15_H_24_	204	0.60
7	17.65	γ-muurolene	sesquiterpene	C_15_H_24_	204	0.73
8	17.77	humulene	sesquiterpene	C_15_H_24_	204	1.15
9	18.40	germacrene-D	sesquiterpene	C_15_H_24_	204	22.01
10	18.76	bicyclogermacrene	sesquiterpene	C_15_H_24_	204	2.80
11	25.97	phytol	diterpene alcohol	C_20_H_40_O	296	2.22
12	42.05	squalene	triterpene	C_30_H_50_	410	1.30
13	48.41	palmitone	ketone	C_31_H_62_O	450	16.92

### The antioxidant activity of *A. squamosa* extracts

#### Free radical scavenging activity

**Fig. 2:**
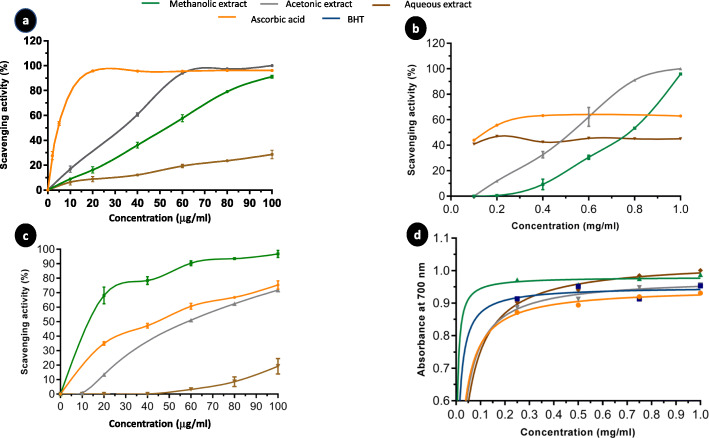
The antioxidant activity of *Annona squamosa* leaves extracts. **a** The dose-dependent DPPH scavenging activity of leaves extracts relative to that of L-ascorbic acid. **b** The dose-dependent hydrogen peroxide scavenging activity of leaves extracts relative to that of L-ascorbic acid. **c** The dose-depended nitric oxide radical scavenging activity of leaves extracts. Gallic acid and L-ascorbic acid were used as standards. **d** The reducing power assay of different concentrations of leaves extract, L-ascorbic acid, and BHT. All results are presented as the mean ± SD (*n* = 3)

*A. squamosa* leaves extracts were tested for their ability to scavenge the DPPH radical, as DPPH is one of the few stable and commercially available organic nitrogen radicals [28]. As shown in Fig. 2-a, the studied extracts displayed dose-dependent DPPH scavenging activities in the following order: acetone extract > methanol extract > water extract. Furthermore, the concentrations of the extracts that were able to scavenge 50% of the DPPH radical (IC_50_) were calculated and are presented in Table 1. The acetone extract had the lowest IC_50_ (33.9 ± 4.8 μg/mL), followed by methanol (IC_50_ = 51 ± 1.6 μg/mL) and then water (IC_50_ = 98.3 ± 0.4 μg/mL) extracts.

#### H_2_O_2_ scavenging activity

The ability of *A. squamosa* extracts to scavenge H_2_O_2_ was investigated in relation to that of ascorbic acid; the results are shown in Fig. 2-b. Our findings demonstrated that the *A. squamosa* extracts exhibited dose-dependent scavenging of H_2_O_2_. The water extract exhibited the most efficient H_2_O_2_-radical scavenging ability, with an IC_50_ value of 110 ± 14.1 μg/mL (vs. ascorbic acid, IC_50_ = 55 ± 7.1 μg/ml), followed by methanolic (IC_50_ = 735 ± 49.5 μg/mL) and acetonic (IC_50_ = 516 ± 5.8 μg/mL) extracts (Table 1).

#### NO scavenging activity

The NO scavenging activity of *A. squamosa* extracts was determined by following the decrease in the absorbance at 546 nm, as described by Boora et al. [29], and the results were presented as a percentage of scavenging activity in Fig. 2-c. All extracts possessed dose-dependent NO scavenging activity. The methanolic extract was found to be more efficient than the reference, ascorbic acid, with IC_50_ values of 12 ± 4.2 μg/mL and 16.5 ± 2.12 μg/mL, respectively. The acetone and water extracts were less effective, with IC_50_ values of 44 ± 5.7 μg/mL and 81 ± 1.4 μg/mL, respectively (Table 1).

#### Reducing power of *A. squamosa* leaves extracts

The reducing power of *A. squamosa* extracts was evaluated by the reduction of Fe^3+^ to Fe^2+^ and compared with ascorbic acid and BHT, as standard references. The data in Fig. 2-d demonstrate that reducing power increased as the concentration of the extract increased. At 0.75 mg/mL, the absorbance values of *A. squamosa* extracts were higher than the standards and followed the order: water extract (0.984) > methanol extract (0.975) > acetone extract (0.95) > ascorbic acid (0.92) > BHT (0.91).

### The anticancer activity of *A. squamosa* extract on colon cancer cell lines

#### The effect of *A. squamosa* extract on cell morphology and survival

**Fig. 3:**
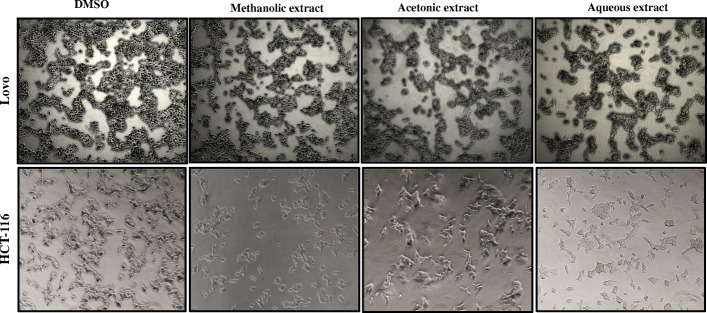
The effect of *A. squamosa* extracts on the morphology of colon cancer cell lines. Lovo and HCT-116 cells were seeded at 4 × 10^5^ and stimulated with 50 μg/ml methanol, acetone or water extract in DMEM medium with 10% FBS. Photomicrographs were taken at 24 h

**Fig. 4:**
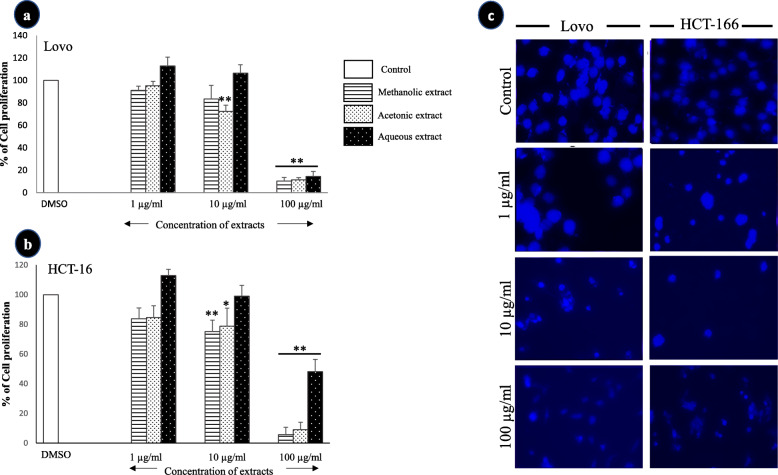
The effect of *A. squamosa* extracts on the proliferation of colon cancer cell lines as determined by using MTT and nucleus staining assays. The cell viability of Lovo cells (**a**) and HCT-116 cells (**b**) in the MTT assay (*n* = 5). **c** Photomicrographs of colon cell lines treated with three different concentrations of methanolic extract of *A. squamosa* leaves. The control comprised cells treated with DMSO (*p < 0.05, **p < 0.005)

The treatment of different colon cancer cell lines with *A. squamosa* leaves extracts did not induce noticeable changes in cell morphology, but it affected the number of cells (Fig. 3). Therefore, the anti-proliferative activity of the extracts was evaluated using an MTT assay. The data in Fig. 4 demonstrate that the tested extracts (methanolic, acetonic, and aqueous) inhibited the proliferation of colon cancer cells in a dose-dependent manner. All extracts induced a highly significant decrease in Lovo cell viability at 100 μg/mL, with more than 85% inhibition (Fig. 4-a). Similarly, at the same concentration, the methanolic, acetonic, and aqueous extracts inhibited HCT-116 cell proliferation by 94, 91, and 58%, respectively (Fig. 4-b). Furthermore, to confirm the antiproliferative activity of the *A. squamosa* extracts, nuclear staining was performed. The treatment of the cell lines with different concentrations of methanolic extract (1, 10, and 100 μg/mL) resulted in a marked dose-dependent decrease in the number of cells and notable damage to the nucleus (Fig. 4-c). A similar effect was observed after treatment acetone and water extracts (data not shown).

#### The toxicity of *A. squamosa* extracts to cells

**Fig. 5:**
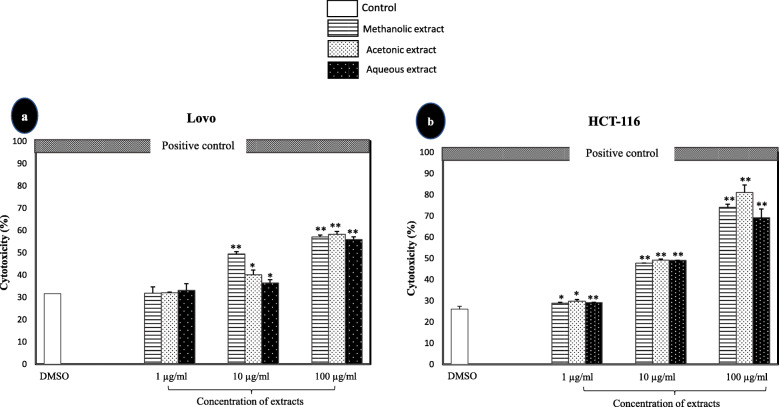
The cytotoxic effect of *A. squamosa* extracts against colon cancer cell lines using the LDH assay. Lovo (**a**) and HCT-116 (**b**) colon cancer cell lines. DMSO and 10× Triton were used as negative and positive controls, respectively (*p < 0.05, **p < 0.005)

The toxicity of *A. squamosa* leaves extracts in human colon cancer cell lines was evaluated through the measurement of LDH leakage from degraded cells. As shown in Fig. 5, the cytotoxicity significantly increased with increasing extract concentrations in both tested colon cancer cell lines. The highest concentration of *A. squamosa* extracts tested (100 μg/mL) was 55–58% cytotoxic to Lovo cells. Correspondingly, the cytotoxicity of *A. squamosa* leaves extracts was more apparent in HCT-116 cells; approximately 49% cytotoxicity was induced by 10 μg/mL of the extracts, whereas 100 μg/mL of the methanolic, acetonic, and aqueous extract was 74, 81, and 69% cytotoxic, respectively.

#### The effect of *A. squamosa* extracts on cell migration

**Fig. 6:**
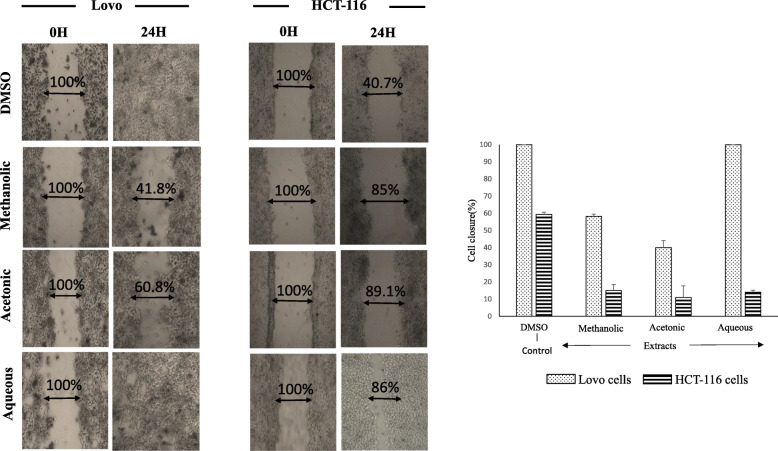
The migration of colon cancer cell lines (Lovo and HCT-116) after treatment with different *A. squamosa* extracts. A scratch was made on each monolayer, the culture medium was refreshed, and 50 μg/mL of extract was added. The cultures were maintained under the appropriate conditions, observed, and photographed at 0 and 24 h (*n* = 3, ^*^p < 0.05, and ^**^p < 0.005)

To clarify the role of *A. squamosa* extracts as potential anticancer agents, their impact on the migration of cancer cells were tested. Overall, treated colon cells showed less migration after 24 h than untreated cells (Fig. 6). Lovo cells treated with methanol or acetone extract showed 41.8–95.8% less closure after 24 h than the control cells (DMSO; *p* < 0.005), and water-extract treated cells were fully migrated. Similarly, the migration of treated HCT-116 cells was significantly lower than the control cells, with 45–49% less migration observed (*p* < 0.005).

### The effect of *A. squamosa* extracts on bacterial growth

The antibacterial activity of the different *A. squamosa* extracts was evaluated through the measurement of the diameter of the inhibition zones surrounding the wells after incubation for 24 h incubation with gram-positive (*B. subtilis*, *S. aureus*, and *E. faecalis*) and gram-negative bacteria (*E. coli, P. aeruginosa*, *K. pneumoniae*, and *LT2)*. As shown in Table 3, the acetone extract displayed antibacterial activity against both gram-positive and gram-negative bacterial strains, except *S. aureus*. However, the methanolic extract inhibited the growth of all the tested bacteria. Moreover, it was the only extract with marked antibacterial activity against *S. aureus* (zone of inhibition = 16.5 ± 0.5 mm), and it was more efficient than the standard antibiotic, tetracycline (zone of inhibition = 14.8 ± 04 mm). The water extract was more sensitive to gram-negative bacteria than gram-positive bacteria, with a moderate antibacterial effect observed only against *E. faecalis* (9.5 ± 0.5 mm). Acetonic extract exhibited 1.3 fold higher antibacterial activity against *P. aeruginosa* than the standard antibiotic.

**Table 3:** Antimicrobial activity of *A. squamosa* extracts. Data are represented as mean of inhibition zone ± SD (n = 3); DMSO and Tetracycline (30 μg) were used negative and positive controls, respectively

Extracts	Zone of inhibition (mm)						
	Gram positive bacteria			Gram negative bacteria			
	*B. subtilis*	*S. aureus*	*E. faecalis*	*E. coli*	*P. aeruginosa*	*K. pneumoniae*	*LT2*
Acetone	11.5 ± 0.5	0.0	8 ± 0	12.6 ± 0.6	24.6 ± 0.6	11.7 ± 0.6	11.5 ± 0.5
Methanol	15.6 ± 0.6	16.5 ± 0.5	7.9 ± 0.1	15.5 ± 0.5	18.3 ± 0.6	11.7 ± 0.6	12.3 ± 1.5
Water	0.0	0.0	9.5 ± 0.5	11.6 ± 0.5	17 ± 1	10.5 ± 0.5	12.2 ± 0.3
DMSO	0.0	0.0	0.0	0.0	0.0	0.0	0.0
Tetracycline	16.2 ± 0.8	14.8 ± 0.4	12.7 ± 0.4	19.3 ± 1.5	18.7 ± 0.6	18.5 ± 0.4	14.3 ± 0.3

## Discussion

Plants with a long history of use in traditional medicine represent a vast resource for the discovery and investigation of new remedies by pharmaceutical sciences [5, 30]. For example, *A. squamosa* has been used extensively in traditional medicine in India, China, and Middle Eastern countries, but the chemical and pharmacological characterization lies behind other species from the same genus, such as *A. muricata* and *A. reticulata*. Several studies have reported the biological activities of different *A. squamosa* parts; fewer have investigated the leaves [18]. The purpose of the current study was to explore the biological activities of *A. squamosa* leaves and examine their use as potential complementary medicine or new remedies to treat diseases, especially cancer.

In phytoscience, one of the most critical challenges faced by researchers is that a single plant contains many bioactive compounds [31]. The identification of the bioactive compounds and their biological activities will help elucidate the toxicity and side effects, calculate the appropriate dosages, and find the best method to extract them. As the successful prediction of botanical compounds from plant material is mostly dependent on the type of solvent used in the extraction procedure [32, 33], three different solvents were used for extraction in this study, and the water extract was found to have the highest yield (Table 1). Thirteen compounds were identified using GC-MS in the methanolic extract of *A. squamosa* leaves. The major compounds were germacrene-D and trans-caryophyllene, which were found to exert different biological activities in a literature review (Fig. 1): for example, germacrene-D was reported to induce antibacterial [34, 35], antioxidant [36], and anticancer activities [36–38]; trans-caryophyllene was shown to exhibit anti-inflammatory [39] and antibacterial activities [40, 41].

OS has been suggested as the root cause of several pathophysiological conditions, including cancer and inflammation [42, 43]. As reported in many studies, several bioactive compounds from plants have shown antioxidant and radical scavenging activity, and a relationship between the antioxidant activity and phenolic content has been reported [44, 45]. In this study, the antioxidant potential of different extracts of *A. squamosa* leaves was determined through the measurement of the scavenging activity and reducing power of RONS (Fig. 2). The DPPH free radical scavenging model demonstrated that acetone extracts displayed the highest scavenging activity, followed by methanol and water extracts (Table 1). Surprisingly, water extracts showed higher H_2_O_2_-scavenging activity than ascorbic acid, followed by the moderate scavenging activity of acetone and methanol extracts; this result agreed with El-Chaghaby et al. [46] (Table 1). Besides, the methanolic extract of *A. squamosa* leaves exhibited good NO-scavenging ability. The overall results agreed with those obtained by Kalidindi et al. [47] and Shirwaikar et al. [48] who investigated the ability of the ethanol, methanol, chloroform, and aqueous extracts of *A. squamosa* leaves to scavenge DPPH, NO, and H_2_O_2_. Several studies revealed correlations between the antioxidant activity and the reducing power of some plant extract [49, 50]. Our findings showed that *A. squamosa* leaves extracts possessed a high reducing power. Interestingly, the methanolic extract exhibited higher reducing power than ascorbic acid and BHT, which were used as reference materials (Fig. 2-d). These results were consistent with previous data reported by Kalidindi et al. [47] and El-Chaghaby et al. [46]. However, the methanolic extract displayed the highest reducing power in both previous studies but was still lower than the standards. This may be as a result of the use of heat in the different extraction methods, which was avoided in our study. Indeed, several studies suggested that heat can affect the stability of various bioactive compounds in plants, especially the flavonoids and phenols, which are strongly correlated with antioxidant activity [51, 52].

In recent decades, as the limitations of cancer treatment strategies have been discovered, researchers have attempted to identify natural products capable of selectively modulating different mechanisms, targeting multiple pathways involved in cancer, and increasing patient survival. However, few studies have investigated the activity of *A. squamosa* extracts as an anticancer agent. In the current study, the anticancer activities of *A. squamosa* leaves extracts were investigated in two colon cancer cell lines, Lovo and HCT-116. Our results revealed that different *A. squamosa* leaves extracts induced cytotoxicity and inhibited the proliferation of both selected cancer cell lines in a dose-dependent manner (Fig. 4). The antiproliferative activity and cytotoxicity of *A. squamosa* leaves extracts were confirmed by nuclear staining, which indicated damaged nuclei (Fig. 4-c). This result suggested that *A. squamosa* leaves extracts inhibited cancer cell proliferation through the induction of apoptosis via caspase-3 activation and cell cycle arrest. Several studies have demonstrated that, in general, plant extracts inhibit cell proliferation by triggering a series of signaling pathways via the phosphorylation of cell cycle proteins, such as MAPK, p53, and EGFR phosphorylation [53, 54]. Indeed, cancer cells’ ability to metastasis and invasion is one of the hallmarks of cancer, and it is considered the leading cause of death among cancer patients [55]. Furthermore, previous studies have shown that Lovo and HCT-116 were highly metastatic [56, 57]. Our results provided the first demonstration that the methanolic or acetonic extracts could partially inhibit cell migration. It is of note that no previous study has investigated the anti-migration activity of any part of the *A. squamosa* plant. However, Zorofchian Moghadamtousi et al. [57] demonstrated that the ethyl acetate extract of *A. muricata* leaves conspicuously blocked the migration of HCT-116 cells, suggesting the similar chemical composition of the leaves of both species (*A. muricata* and *A. squamosa*). A more recent study by Pinto [58] investigated the effects of *A. squamosa* seeds and leaves extracts on different tumor and non-tumor cells’ ability to form colonies. The authors reported that both extracts ultimately reduced the colonogenic survival of MCF-7 and HCT-116 cells, while both extracts showed lower activity against non-tumor cells (VERO). This remarkable anticancer activity may due to the high level of germacrene-D which exerted anticancer activity against different cell lines [36–38], or to the presence of other bioactive compounds which have also been known to have an anticancer activity such as humulene, phytol and/or a combination of these bioactive compounds [59, 60]. Collectively, these findings may assist the future development of novel drugs for colon cancer therapy.

The studies of gut microbiota in normal and pathogenic conditions have demonstrated an association between dysbiosis and human colorectal cancer. Different parts of *A. squamosa* have been used as antimicrobial agents in traditional medicine. The current study demonstrated that different extracts of *A. squamosa* leaves exhibited a broad spectrum of antibacterial activity against both gram-positive and gram-negative bacteria (Table 3). The potent antibacterial activity of *A. squamosa* leaves extracts was recorded against *P. aeruginosa*, a resistant gram-negative bacterial strain. Similarly, Kotkar et al. [61] found that flavonoids isolated from the aqueous extract of *A. squamosa* exerted antibacterial activity against *Pseudomonas, Bacillus*, and *Aspergillus* species. Interestingly, *A. squamosa* leaves extract displayed higher antibacterial activity against the human pathogenic *S. aureus* strain than antibiotics. Indeed, infections caused by *S. aureus* have reached epidemic proportions globally owing to its strong multi-resistance [62]. These results were consistent with those of Pinto et al. [58] who found the significant antibacterial activity of the methanolic extract of *A. squamosa* leaves and seeds against *S. aureus*, *K. pneumoniae*, and *E. faecalis* strains. Vijayalakshmi and Nithiya evaluated the antibacterial activity of *A. squamosa* fruit extracts, which exhibited more potent activity against gram-negative bacteria than gram-positive bacteria. This result could be attributed to several bioactive compounds known to have an antibacterial activity such as benzoquinoline alkaloid, annoquinone, β bourbonene, trans-Caryophyllene, bicyclogermacrene, Palmitone and germacrene-D [19, 40, 63].

## Conclusion

In conclusion, his study attempted to elucidate the ethnopharmacological uses of *A. squamosa* leaves and determine some of the main bioactive compounds which might be responsible for these biological activities. *A. squamosa* leaves extracts may contribute to the development of new remedies as an alternative source of antibiotics or for colon cancer therapies. Further studies are necessary to determine the molecular mechanisms which are targeting by *A. squamosa* leaves extracts.

## Data Availability

The datasets used and/or analyzed during the current study are available from the corresponding author on reasonable request.

## Abbreviations

RONS: Reactive oxygen and nitrogen species
TPC: Total phenolic content
TFC: Total flavonoid content
GC-MS: Gas chromatography coupled with mass spectrometry
DPPH: 2,2-diphenyl-1-picrylhydrazyl
DMSO: Dimethyl sulfoxide
NO: Nitric oxide
H_2_O_2_: Hydrogen peroxide
